# *In vivo* aortic elasticity measurement using electrocardiogram-gated computed tomography: validation with *ex vivo* loading test

**DOI:** 10.1093/icvts/ivaf148

**Published:** 2025-08-19

**Authors:** Junki Yokota, Takashi Shirakawa, Kazuo Shimamura, Takayuki Shijo, Koichi Maeda, Takuji Kawamura, Kizuku Yamashita, Toru Ide, Ryota Matsumoto, Ryoto Sakaniwa, Daisuke Yoshioka, Shigeru Miyagawa

**Affiliations:** Department of Cardiovascular Surgery, Osaka University Graduate School of Medicine, Suita, Osaka, Japan; Department of Cardiovascular Surgery, Osaka International Medical and Science Center, Osaka City, Osaka, Japan; Department of Cardiovascular Surgery, Osaka University Graduate School of Medicine, Suita, Osaka, Japan; Department of Cardiovascular Surgery, Osaka University Graduate School of Medicine, Suita, Osaka, Japan; Department of Cardiovascular Surgery, Osaka University Graduate School of Medicine, Suita, Osaka, Japan; Department of Cardiovascular Surgery, Osaka University Graduate School of Medicine, Suita, Osaka, Japan; Department of Cardiovascular Surgery, Osaka University Graduate School of Medicine, Suita, Osaka, Japan; Department of Cardiovascular Surgery, Osaka University Graduate School of Medicine, Suita, Osaka, Japan; Department of Cardiovascular Surgery, Osaka University Graduate School of Medicine, Suita, Osaka, Japan; Department of Cardiovascular Surgery, Osaka University Graduate School of Medicine, Suita, Osaka, Japan; Department of Social Medicine, Osaka University Graduate School of Medicine, Suita, Osaka, Japan; Department of Cardiovascular Surgery, Osaka University Graduate School of Medicine, Suita, Osaka, Japan; Department of Cardiovascular Surgery, Osaka University Graduate School of Medicine, Suita, Osaka, Japan

**Keywords:** aortic aneurysm, ascending aorta, electrocardiogram-gated computed tomography, mechanical properties, noninvasive measurement

## Abstract

**OBJECTIVES:**

Aortic aneurysm size is a key determinant for surgical intervention, but aortic catastrophes can occur before reaching the size criteria, indicating size alone is insufficient for risk assessment. Aortic mechanical properties could be another factor for predicting such catastrophes. However, its estimation in daily clinical settings remains impractical. This study aimed to validate a noninvasive electrocardiogram-gated computed tomography (EGCT)-based method for assessing aortic mechanical properties by comparing its measurements with *ex vivo* tensile testing of resected specimens.

**METHODS:**

We analysed 49 patients who underwent surgical repair of the ascending aorta. The mechanical properties of the aortic wall were assessed using two parameters: elastic modulus (E), representing stiffness and strain energy (SE), reflecting stored deformation energy. *Ex vivo* loading test was performed on resected specimens, while *in vivo* measurements were obtained from preoperative EGCT scans using a custom analysis plugin. Correlation and agreement between methods were evaluated using Spearman’s correlation (r), Bland-Altman analysis and intraclass correlation coefficients (ICC).

**RESULTS:**

EGCT-based measurements of E and SE showed strong correlations with *ex vivo* loading test (*r* = 0.733 and *r* = 0.773, respectively). Bland-Altman analysis demonstrated good agreement, with minimal bias for E and a negative proportional bias for SE. ICC values indicated good-to-excellent reliability for E (0.86) and moderate reliability for SE (0.63).

**CONCLUSIONS:**

EGCT-based measurement is a feasible, reliable method for assessing aortic mechanical properties noninvasively. Although further studies are needed to refine predictive accuracy, this approach may enhance risk stratification for aortic catastrophes in future clinical practice.

## Graphical Abstract

**Figure:**
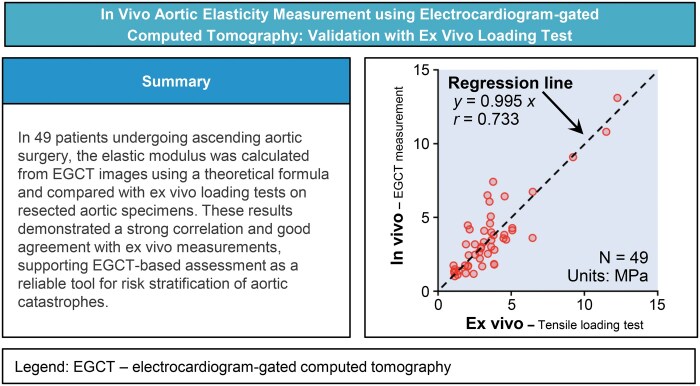

## INTRODUCTION

The size of an aortic aneurysm is believed to have a significant impact on the risk of aortic catastrophes such as dissection or rupture [1–3], which is why current clinical guidelines provide diameter-based criteria for elective surgical intervention [4–6]. However, in actual clinical practice, aortic catastrophes can occur before an aneurysm reaches the dimensional criteria [7]. This discrepancy suggests that size alone may not be sufficient for risk assessment, highlighting the need to evaluate additional biomechanical properties of the aorta [8–10].

Previous studies have directly measured the mechanical properties of the aortic wall using surgically resected specimens, revealing their potential value in assessing aortic risk [11–14]. These properties include tensile strength, elasticity and extensibility, which determine how the aortic wall responds to external forces such as blood pressure and mechanical stress. While these measurements provide important insights, it is inherently impossible to ascertain them prior to surgical resection. Alternatively, noninvasive measurement methods of aortic wall properties in medical images have also been proposed [15, 16]. However, some modalities are not widely used in routine aortic disease management. If a readily available noninvasive method were established, surgeons could assess aortic risk and estimate its progression during outpatient visits based on individual aortic biomechanics, leading to timely interventions.

This study aims to evaluate the feasibility of a noninvasive, *in vivo* method for measuring two mechanical properties, the elastic modulus (E) and strain energy (SE) of the aorta, using electrocardiogram-gated computed tomography (EGCT) in actual surgical cases (see Technical terms). The proposed measurement approach is based on our previously reported theoretical framework [17]. To validate its accuracy, we compared the EGCT-based measurements with the results of an *ex vivo* tensile loading test performed on specimens collected intraoperatively.

### Technical terms

- Elastic modulus (E): A measure of a material’s stiffness. A higher E means the material resists stretching more when pulled or pushed.
- SE: The energy stored in a material due to deformation. It reflects both the applied force (stress) and the resulting deformation (strain).
- Load/force: The strength of the external force applied to an object.
- Stress: Force per unit area, similar to blood pressure acting on vessel walls.
- Displacement: The distance a point on an object moves due to the object’s deformation.
- Strain: The ratio of displacement to the original length, representing how much an object stretches or deforms relative to its initial size.

## METHODS

We employed two methodologies—a loading test of aortic wall specimens and theoretical formulae using EGCT images of aortic cross-sections—to estimate the elastic modulus (E) and the SE of the human aorta (see Technical terms). Both methodologies were used to measure E and SE, and their correlations were analysed to validate the accuracy of the CT-based measurements. Specifically, in this study, E represents the stiffness of the aortic wall within the diastole-to-systole range, while SE represents the deformation energy stored in the aortic wall from diastole to systole.

### Patient selection

The study population consisted of 168 consecutive surgical patients who underwent prosthetic graft replacement for thoracic aortic aneurysms at Osaka University Hospital between September 2020 and June 2022. We excluded patients if tissue samples of the aorta with the entire circumference or complete wall layers could not be obtained during surgery, or if preoperative EGCT images were unavailable, or if they had ascending aortic dissection.

### *Ex vivo* loading test of the aortic wall

**Figure 1:**
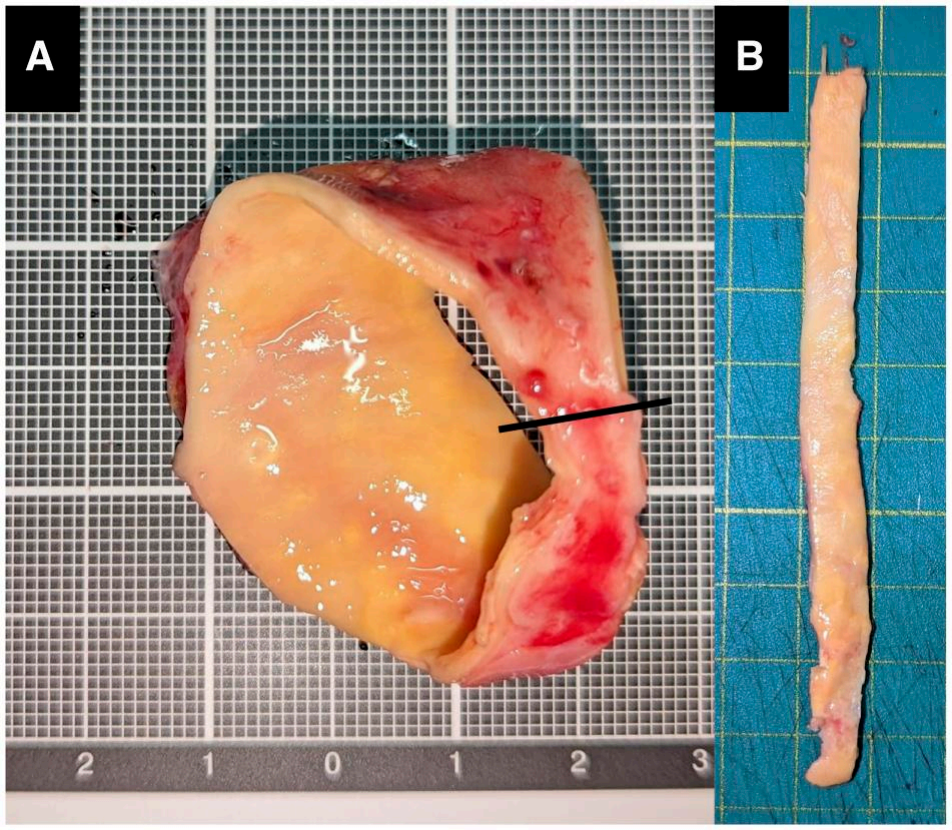
Creation of aortic wall specimen. (**A**) The resected aorta is a short, curved tube or a ring from the middle section of the ascending aorta because surgeons keep safe margins above the sinotubular junction and under brachiocephalic artery. (**B**) We cut and opened it at the small curvature side and then trimmed the sides to make a straight strip with the entire circumferential length of the ascending aorta

The ascending aorta was resected while preserving its circular shape (Fig. 1A). The specimen was then cut at the small curvature side and trimmed into a straight strip (Fig. 1B). The trimming width was adjusted so that applying a 250 g load would correspond to each patient’s systolic blood pressure; for example, with the same 250 g load, a narrower width results in higher tensile stress, corresponding to a higher blood pressure (see Appendix S1 for details). The specimen’s thickness was measured at 10 points, and the median thickness was used for analysis. Tissue strips without prominent calcification or thickening were selected. Each specimen was either tested immediately after resection or stored in acetic acid Ringer’s solution at 4°C and tested within 24 hours.

A custom apparatus was used, consisting of a 40 cm frame with an upper grip to suspend a tissue strip, and a lower grip to apply a tensile load downwards. Five 50 g weights were sequentially added to the 8 g lower grip, reaching a total load of 258 gf. To measure displacement, two 27-gauge needles were inserted at both ends of the strip, and the distance between them was measured using a digital calliper, Mitutoyo ABSOLUTE Coolant Proof Caliper (Mitutoyo Corporation, Kanagawa, Japan). Each test was performed three times per specimen, and the average result was used for analysis.

#### Explanatory note

Using longer tissue strips reduces the impact of measurement error on total deformation. Additionally, since the CT-based measurement described in the following section assesses circumferential properties, we opted for circumferentially oriented long tissue strips for uniaxial tensile testing.

**Figure 2:**
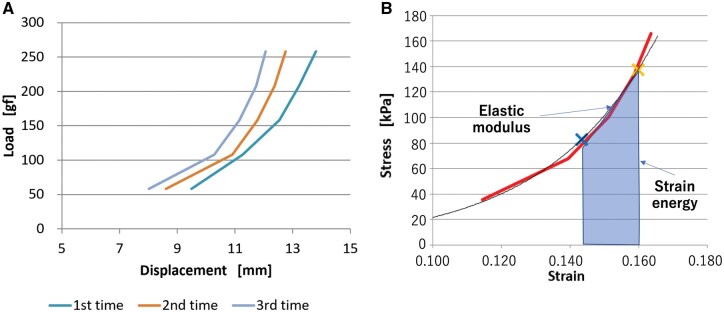
*Ex vivo* loading test of aortic wall specimen. (**A**) An example of load-displacement curves obtained three times from *ex vivo* loading tests for each tissue specimen. (**B**) The stress-strain relation calculated from (A). A red connected line corresponds to the average of three measurements in (A). A black curve indicates the exponential approximate curve. A yellow cross indicates the position corresponding to the patient's systolic blood pressure, and a blue cross indicates diastolic pressure. The elastic modulus of the aortic wall specimen is the slope between those systolic and diastolic points. The strain energy is the area under the curve between them

Load-displacement curves were generated from the above experimental values (Fig. 2A). Stress-strain curves were obtained by dividing load and displacement by the cross-sectional area and initial length, respectively (Fig. 2B). True stress was calculated by considering cross-sectional changes, assuming the aortic wall to be incompressible. The slope between the points corresponding to systolic and diastolic pressures (blue and yellow crosses in Fig. 2B, respectively) represents E of the aortic wall, while the area under the stress-strain curve, fitted with an exponential approximation (black curve in Fig. 2B), represents SE.

### *In vivo* measurement in CT images

**Figure 3:**
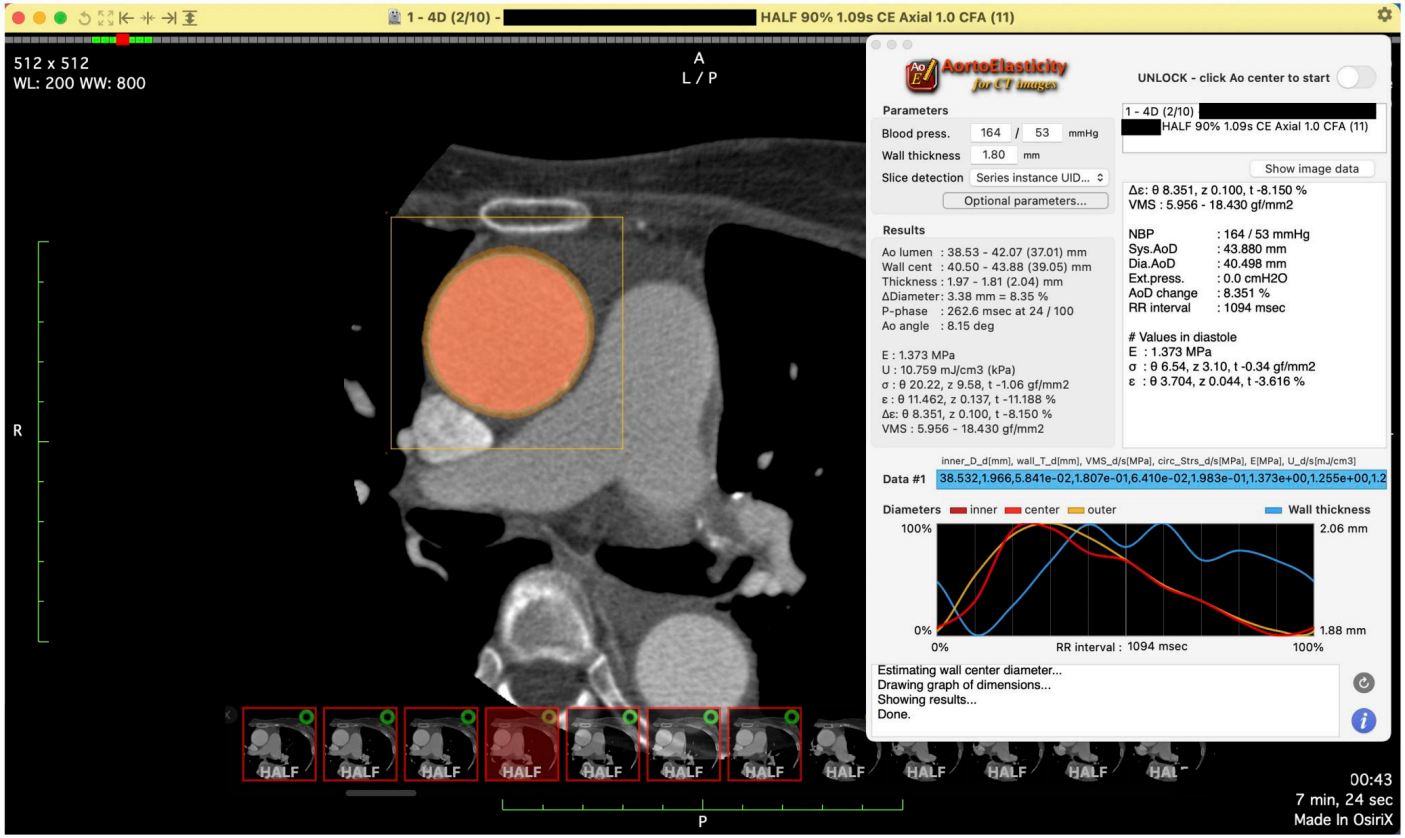
*In vivo* measurement of aortic mechanical properties by EGCT. Theoretical quantification formulae are implemented in our custom plug-in software connected to OsiriX MD (Pixmeo SARL, Bernex, Switzerland), an FDA-certified DICOM viewer. Processes other than the implemented formulae, such as segmentation by region-growing method, depend on the internal functions provided by OsiriX MD. FDA indicates Food and Drug Administration

We performed preoperative EGCT of the aorta (see Table 1 for scan parameters). For measurement, we developed a custom analysis plugin integrated with the DICOM viewer OsiriX MD (Pixmeo SARL, Bernex, Switzerland) (Fig. 3). A cross-section of the ascending aorta was selected midway between the sinotubular junction and brachiocephalic artery, ensuring that calcified or thickened regions were avoided. If the aortic axis was tilted more than 20 degrees, multiplanar reconstruction was used to generate a new orthogonal cross-section.

**Table 1:** CT scanners and protocols

CT scanner	Aquilion ONE	Revolution CT
Detector	320 rows	256 rows
Scan mode	Volumetric scan	Helical scan
ECG gating	Prospective	Retrospective
Collimation	320 × 0.5	256 × 0.625
Slice thickness [mm]	0.5 or 1.0	0.625 or 1.25
Pixel spacing [mm]	0.261–0.390	0.260–0.283
Exposure time [ms]	275	280
Tube voltage [kVp]	120	120
Tube current [mA]	240–750	69–655
Number of patients	37	12
Images per cardiac cycle	10 images (0–90% phases)	

Aquilion ONE (Canon Medical Systems Corporation, Tochigi, Japan). Revolution CT (GE HealthCare Technologies Inc., IL, USA).

The elastic modulus, E, and the strain energy, SE, were calculated at the selected cross-section using the theoretical formulae as reported previously [17]. These formulae were implemented in our analysis plugin. The clinical parameters for applied formulae were obtained as followed.

The aortic diameter was automatically measured using region-growing segmentation. Pulse pressure was determined as noninvasive blood pressure (NIBP) readings taken before CT scanning. Wall thickness was manually measured at five different points in the selected cross-section in diastole by a certified cardiovascular surgeon (Supplementary Fig. S1). Thickness was defined as the distance between the inner border by the contrast-enhanced lumen and the outer border by low CT values of surrounding structures.

### Statistical analysis

The continuous variables without normal distribution were written in median and interquartile range 25–75%, and the binary variables were written in count and percentage.

We used R version 4.4.2 (R Foundation for Statistical Computing, Vienna, Austria) for statistical analysis. We employed Spearman’s rank correlation coefficient to assess the correlation between the measurements of the *ex vivo* loading test and *in vivo* EGCT. We also used Bland-Altman analysis and intraclass correlation coefficient (ICC, two-way random-effects model) to assess the agreement between those measurement methods. Regarding missing data, there was no missing information for the parameters needed for measurement so that missing data imputation was not carried out in background factors.

### Ethical statement

This study was conducted in accordance with the World Medical Association (WMA) Declaration of Helsinki and was approved by the Osaka University Hospital Ethics Committee (approval no. 22026) as a noninterventional study using surplus specimens. Written informed consent for study participation and data publication was obtained from all participants. Data and biological materials are stored in an institutional database, the use of which is approved and monitored by the Ethics Committee in compliance with the WMA Declaration of Taipei.

## RESULTS

### Patient selection

Among 168 consecutive surgical patients, 29 were excluded due to incomplete aortic tissue samples, 48 due to the absence of sufficient preoperative EGCT images and 42 due to ascending aortic dissection. The baseline characteristics of the 49 patients included in this study are presented in Table 2. Among the enrolled patients, 39 had hypertension, 19 had an ascending aorta ≥ 45 mm and 8 had a bicuspid aortic valve. During the study period, there were no surgical cases of connective tissue disease.

**Table 2:** Patient characteristics

Patients	*n* = 49
Age [years]	75.0 (62.5–78.0)
Male	32 (65.3%)
Body mass index [kg/m^2^]	22.3 (20.7–24.3)
Background	
Hypertension	39 (79.6%)
Diabetes mellitus	9 (18.4%)
Dyslipidaemia	22 (44.9%)
Chronic kidney disease	11 (22.4%)
History of smoking	29 (59.2%)
Connective tissue disease	0 (0.0%)
Systolic blood pressure [mmHg]	145 (125–158)
Diameter of ascending aorta in CT [mm]	43.0 (38.0–49.0)
Anatomical characteristics	
Ascending aorta ≥ 45 mm	19 (38.8%)
Annuloaortic ectasia	5 (10.2%)
Aortic arch aneurysm	20 (40.8%)
Stanford type-B aortic dissection	5 (10.2%)
Tricuspid aortic valve	41 (83.7%)
Bicuspid aortic valve	8 (16.3%)

Case count and percentage if no unit specified, or median and interquartile range (25–75%).

### Mechanical properties

The elastic modulus, E, was 3.45 ± 2.28 MPa in the *ex vivo* loading test and 2.90 ± 2.18 MPa in the *in vivo* EGCT measurement. The strain energy, SE, was 7.03 ± 4.45 and 4.31 ± 2.38 kJ/m^3^, respectively.

#### Regression analysis

**Figure 4:**
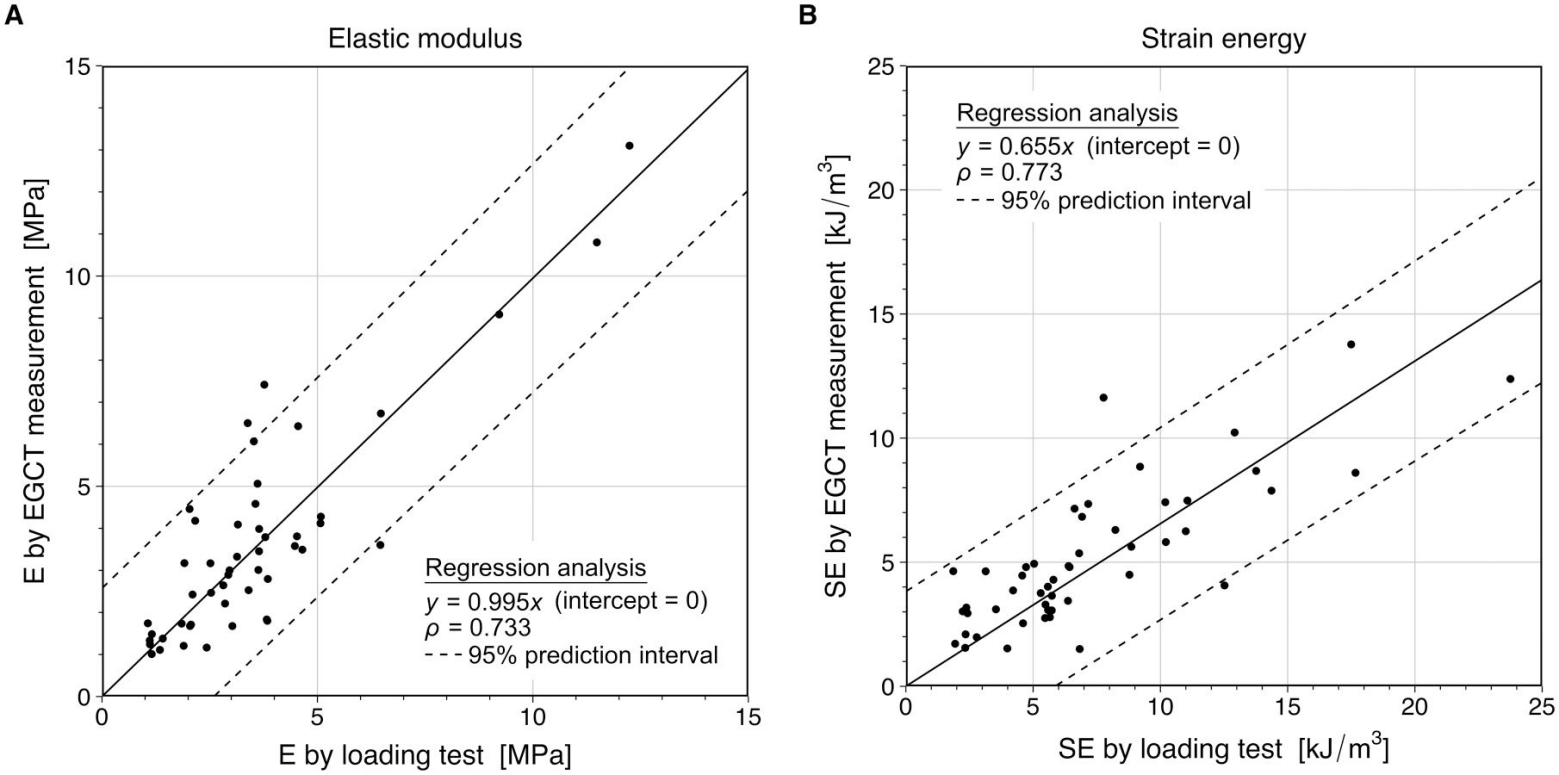
Regression analysis between loading test and EGCT measurement. Correlations and regression lines between the *ex vivo* loading tests and *in vivo* EGCT measurements for (**A**) the elastic modulus and (**B**) strain energy of 49 cases. Linear regression lines (solid lines) were fitted with the intercepts fixed at the origin. Dashed lines indicate 95% prediction interval. EGCT indicates electrocardiogram-gated computed tomography; E, elastic modulus; SE, strain energy

A linear correlation was observed between the loading test and CT-based measurement for both E and SE (Fig. 4). Spearman’s rank correlation coefficients (r) were 0.733 and 0.773, respectively, indicating a strong positive correlation.

#### Bland-Altman analysis

**Figure 5:**
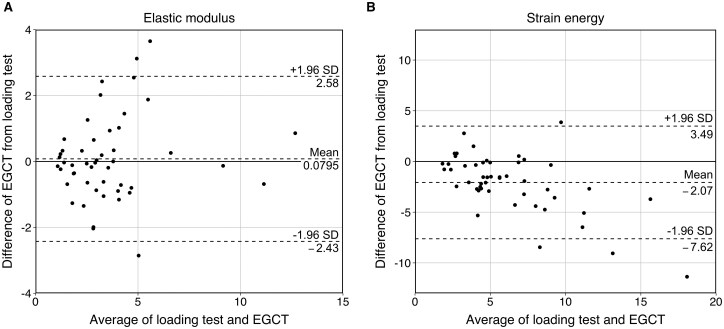
Bland-Altman plot for loading test and EGCT measurement. Bland-Altman plot for the *ex vivo* loading tests and *in vivo* EGCT measurements for (**A**) the elastic modulus and (**B**) strain energy of 49 cases. Dashed lines indicate the mean and 95% limits of agreement, represented by ±1.96 SD. EGCT indicates electrocardiogram-gated computed tomography; SD, standard deviation

We evaluated the agreement between the loading test and CT-based measurement for E and SE using Bland-Altman plots (Fig. 5).

- For E, 46 out of 49 cases (93.9%) fell within the limit of agreement. The bias was minimal (0.0795) and showed no proportional trend.
- For SE, 45 out of 49 cases (91.8%) were within the limit of agreement. However, a negative proportional bias was observed, suggesting that EGCT measurements may not align with the loading test in the higher SE range.

#### Intraclass correlation coefficient

We calculated the ICC using a two-way random-effects model for absolute agreement, which assesses the agreement between CT-based and *ex vivo* measurements.

- For E, the ICC was 0.86 (95% CI: 0.77–0.92), indicating good to excellent reliability.
- For SE, the ICC was 0.63 (95% CI: 0.24–0.82), indicating moderate reliability.

Supplementary Table S1 presents individual measurements for each case. Supplementary Table S2 shows measurements categorized by patient subgroups, providing preliminary data for smaller patient groups.

## DISCUSSION

### Previous studies

The mechanical properties of the aortic wall were first reported in the 1880s [18]. Since then, elasticity, distensibility and stiffness have been assessed [19] and measured in animals [20], cadavers [21] and surgical specimens [11–14]. These properties have been linked to aortic catastrophes, gaining recognition in both bioengineering and surgical research [22–25]. While noninvasive methods are essential for assessing aortic strength and compliance [15, 16], their complexity often hinders their integration in routine clinical practice. The stiffness parameter β [26] and distensibility [27], which are defined as the ratio of diameter change to pulse pressure during a cardiac cycle, are relatively simple evaluation methods. Although both parameters demonstrated moderate correlations with the experimental data (*r* = 0.660 for β and *r* = 0.616 for distensibility, Supplementary Fig. S2), our method demonstrated a higher correlation, further supporting its validity. Pulse wave velocity (PWV) is another indicator of arterial compliance. However, the squared brachial-ankle PWV (baPWV) measured in 45 cases with ankle-brachial index ≥ 0.8 in this study showed a relatively weak correlation with the experimental data (*r* = 0.329). This discrepancy is likely due to baPWV reflecting the characteristics of peripheral arteries from the upper arm to the ankle, whereas our study evaluates local aortic elasticity.

### Towards implementation for clinical analysis

Our results demonstrated a strong correlation between *ex vivo* tensile testing and *in vivo* CT-based measurement for both E and SE in regression analysis. Bland-Altman analysis confirmed the reliability of CT-based measurement for E, while a proportional bias was observed for SE. The ICC showed good to excellent reliability for E, while SE exhibited moderate reliability. Given this strong correlation, applying adjustments such as a proportional correction factor for SE may improve measurement accuracy.

This study suggests that CT-based measurement methods enable the assessment of aortic wall mechanical properties without the need for surgical specimen extraction, making it feasible to implement these evaluations as routine examinations. This advancement could facilitate the following applications in clinical research and practice:

- Large-scale data collection: The ability to noninvasively measure aortic mechanical properties across a wide range of cases may accelerate research on the relationship between aortic wall mechanics, disease progression, and patient prognosis.
- Pathological analysis: If correlations between CT-derived mechanical properties and histopathological markers such as elastin content can be established, CT imaging may serve as a tool for classifying and analysing individual pathological backgrounds noninvasively.
- Aortic screening: CT-based aortic check-ups may be integrated into routine health examinations, allowing for early detection of high-risk cases.
- Personalized risk stratification: By identifying patients based on individual aortic biomechanics, physicians could proactively monitor those at increased risk of dissection or rupture, leading to timely interventions.
- Relations with other physiological tests: Physiological tests, such as PWV, reflect arteriosclerosis, while CT-derived mechanical properties may evaluate aortosclerosis. Their combination could enhance overall arterial risk assessment.

However, E and SE are not the sole indicators of mechanical state—factors like wall thickness, displacement and tensile stress also play a role. It remains unclear which mechanical properties best predict aortic catastrophes. A deeper understanding of aortic disease may come from combining imaging and physiological tests, including CT.

### Preliminary analysis of patient-specific characteristics

We compared measurements across clinical subgroups based on age, sex, aortic diameter, wall thickness, systolic blood pressure and anatomical characteristics (Supplementary Table S2). Due to the limited sample size, definitive conclusions cannot be drawn from this table. However, our analysis of larger cohorts with only EGCT measurements has demonstrated a consistent trend: an increase in aortic diameter and wall thinning is associated with a higher elastic modulus. Similarly, ageing is expected to correlate with an increase in elastic modulus, but the degree of this effect varies among individuals. Given the sample size of this study, this association could not be clearly detected. Additionally, the statistical power was insufficient to assess the impact of other patient characteristics. Future studies incorporating large-scale clinical data will enable more detailed and statistically robust analyses.

### Limitations

No enrolled patients exhibited specific findings of connective tissue diseases. Uniaxial and biaxial tensile testing are commonly used to assess aortic mechanical properties. In this study, uniaxial testing was selected to ensure measurement accuracy with minimal error, though it may not fully replicate the physiological three-dimensional conditions. Tissue specimens were either tested immediately after resection or stored in acetic acid Ringer’s solution at 4°C and tested within 24 hours. It is unclear whether the difference in specimen handling affects the results. The theoretical formulae for EGCT measurement assume a homogeneous circular tube; however, aortic specimens exhibit heterogeneity, including atherosclerotic lesions and varying wall thicknesses. The formulae do not consider the viscosity of the aortic wall. Central blood pressure was not considered, as NIBP values were used. Since NIBP is typically lower than central blood pressure, this may have influenced our measurements. Our results showed a negative proportional bias in SE, indicating slightly lower SE values in the CT method compared to tensile testing.

## CONCLUSIONS

We measured the E and SE of the aorta of surgical patients. The *in vivo* EGCT-based measurements, derived from the reported theoretical formulae, demonstrated a strong positive correlation with the *ex vivo* measurements obtained by the tensile loading tests of surgically resected specimens. This method has the potential to assess the mechanical properties of the aorta noninvasively, which will contribute to revealing high-risk patients with aortic catastrophes in future clinical practice.

## Supplementary Material

ivaf148_Supplementary_Data

## Data Availability

The data underlying this article are available in Osaka University Knowledge Archive at https://doi.org/10.60574/97677.

## ABBREVIATIONS

E: Elastic modulus
EGCT: Electrocardiogram-gated computed tomography
SE: Strain energy
