# Supplementary material for: In vivo aortic elasticity measurement using electrocardiogram-gated computed tomography: validation with ex vivo loading test
Source: Interdiscip Cardiovasc Thorac Surg. 2025 Aug 19;40(8):ivaf148. doi: 10.1093/icvts/ivaf148 (PMC12375406; doi:10.1093/icvts/ivaf148)
Supplement: ivaf148_Supplementary_Data [file ivaf148_supplementary_data.zip › Supplemental_figure/Supplementary Figure 1.pdf]

Supplementary Figure 1. Cross section of the aorta in each case.

The thickness of the aortic wall was measured at five points (colored letters) in diastole of each case and the mean value was taken as the thickness of the case.

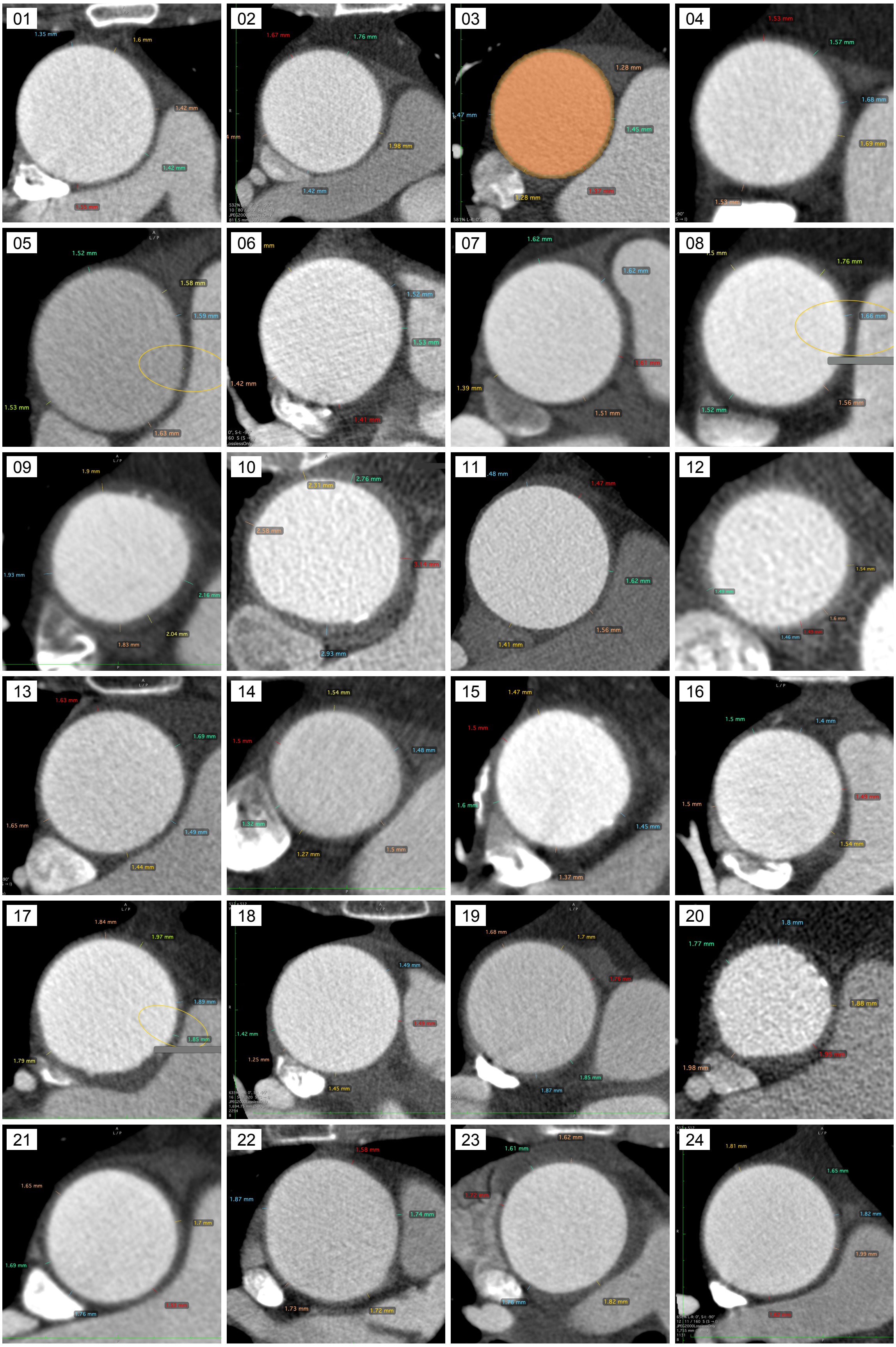

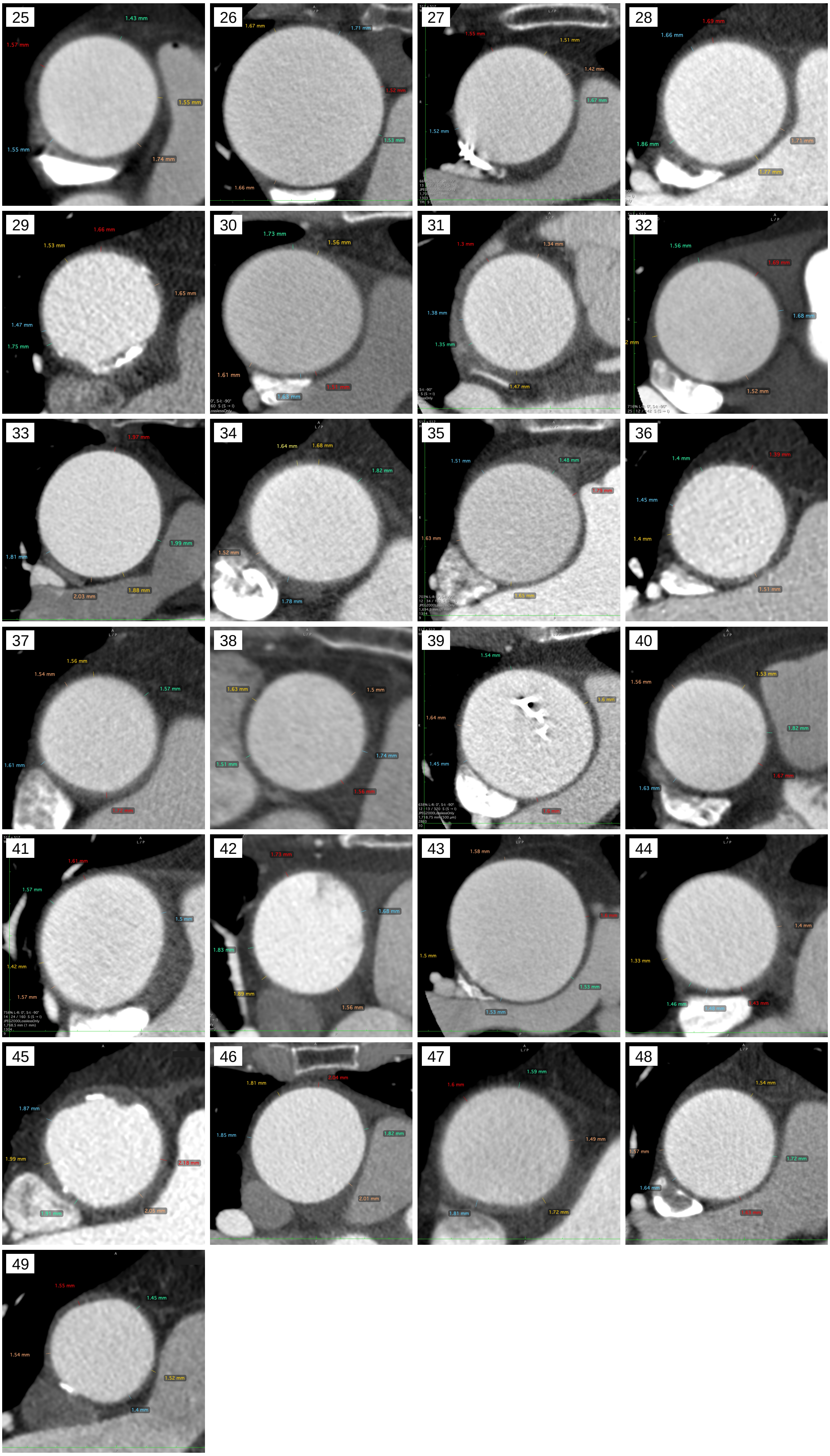
25
26
27
28
29
30
31
32
33
34
35
36
37
38
39
40
41
42
43
44
45
46
47
48
49
