# Supplementary material for: In vivo aortic elasticity measurement using electrocardiogram-gated computed tomography: validation with ex vivo loading test
Source: Interdiscip Cardiovasc Thorac Surg. 2025 Aug 19;40(8):ivaf148. doi: 10.1093/icvts/ivaf148 (PMC12375406; doi:10.1093/icvts/ivaf148)
Supplement: ivaf148_Supplementary_Data [file ivaf148_supplementary_data.zip › Supplemental_figure/Supprementary table 1.docx]

Supplementary Table 1. Measurements of each case

| Age | Sex | Systolic blood pressure  [mmHg] | Aortic diameter  [mm] | Loading test | | | | EGCT | | |
| --- | --- | --- | --- | --- | --- | --- | --- | --- | --- | --- |
|  |  |  |  | Specimen width  [mm] | Specimen thickness *1  [mm] | Elastic modulus  [MPa] | Strain energy  [kJ/m^3^] | Aortic wall thickness *2  [mm] | Elastic modulus  [MPa] | Strain energy  [kJ/m^3^] |
| 41 | F | 144 | 42.6 | 6.00 | 1.22 (1.12 – 1.26) | 2.07 | 17.7 | 1.47 | 1.71 | 8.60 |
| 55 | M | 155 | 55 | 4.31 | 1.40 (1.25 – 1.51) | 11.5 | 5.66 | 1.73 | 10.8 | 2.79 |
| 72 | F | 140 | 44 | 5.97 | 1.47 (1.33 – 1.72) | 3.85 | 6.63 | 1.37 | 2.80 | 7.16 |
| 75 | F | 132 | 35 | 7.96 | 1.54 (1.42 – 1.60) | 3.52 | 3.99 | 1.60 | 6.07 | 1.53 |
| 78 | M | 118 | 46.5 | 6.70 | 1.37 (1.29 – 1.49) | 3.82 | 7.16 | 1.57 | 1.83 | 7.35 |
| 52 | F | 155 | 48.2 | 4.92 | 1.22 (1.15 – 1.30) | 3.84 | 12.9 | 1.46 | 1.80 | 10.2 |
| 76 | M | 123 | 46.5 | 6.43 | 1.52 (1.39 – 1.58) | 3.63 | 6.43 | 1.55 | 3.01 | 4.80 |
| 62 | M | 122 | 36.5 | 8.26 | 1.72 (1.59 – 1.86) | 1.34 | 5.30 | 1.60 | 1.11 | 3.76 |
| 76 | M | 151 | 35.5 | 6.86 | 2.14 (1.88 – 2.34) | 1.90 | 4.21 | 1.97 | 1.21 | 3.86 |
| 78 | M | 166 | 45 | 4.92 | 1.84 (1.59 – 2.45) | 3.61 | 6.83 | 2.74 | 5.06 | 1.50 |
| 78 | M | 126 | 45 | 6.49 | 1.57 (1.54 – 1.60) | 3.13 | 5.80 | 1.51 | 3.33 | 4.29 |
| 63 | M | 126 | 31 | 9.42 | 1.68 (1.57 – 1.72) | 1.15 | 6.39 | 1.52 | 1.01 | 4.85 |
| 68 | F | 164 | 41.8 | 5.37 | 1.27 (1.14 – 1.34) | 3.03 | 17.5 | 1.58 | 1.68 | 13.8 |
| 76 | M | 136 | 35 | 7.73 | 1.89 (1.76 – 2.03) | 3.77 | 2.33 | 1.47 | 7.42 | 1.55 |
| 77 | M | 176 | 37 | 5.65 | 1.64 (1.46 - 2.07) | 4.55 | 5.59 | 1.48 | 6.43 | 3.07 |
| 69 | F | 139 | 43 | 6.15 | 1.62 (1.45 – 1.65) | 2.96 | 6.81 | 1.49 | 3.00 | 5.36 |
| 85 | M | 150 | 43 | 5.70 | 1.86 (1.62 - 1.99) | 3.56 | 5.73 | 1.87 | 4.58 | 3.06 |
| 53 | F | 158 | 50 | 4.66 | 1.36 (1.28 – 1.55) | 2.52 | 23.8 | 1.42 | 3.17 | 12.4 |
| 71 | M | 152 | 50 | 4.84 | 1.76 (1.70 - 1.80) | 3.15 | 8.78 | 1.77 | 4.09 | 4.49 |
| 65 | M | 105 | 43 | 8.15 | 2.68 (2.36 – 2.71) | 1.16 | 3.54 | 1.88 | 1.48 | 3.10 |
| 78 | M | 169 | 35 | 6.22 | 2.06 (1.96 - 2.15) | 2.16 | 5.73 | 1.67 | 4.18 | 3.65 |
| 25 | F | 96 | 53 | 7.23 | 2.41 (2.32 – 2.58) | 2.04 | 3.14 | 1.66 | 1.68 | 4.63 |
| 27 | M | 94 | 50 | 7.83 | 2.41 (2.29 - 2.54) | 2.82 | 2.38 | 1.71 | 2.65 | 3.17 |
| 60 | M | 140 | 50 | 5.25 | 1.74 (1.56 – 1.93) | 1.85 | 13.8 | 1.78 | 1.73 | 8.68 |
| 79 | M | 183 | 39 | 5.15 | 1.63 (1.46 - 1.66) | 3.79 | 8.23 | 1.57 | 3.79 | 6.30 |
| 79 | F | 130 | 70 | 4.04 | 1.70 (1.55 – 1.85) | 12.2 | 2.78 | 1.62 | 13.1 | 1.98 |
| 78 | F | 168 | 46 | 4.76 | 1.33 (1.24 - 1.43) | 5.07 | 10.2 | 1.53 | 4.12 | 5.81 |
| 77 | M | 103 | 39 | 9.16 | 1.92 (1.82 – 2.25) | 1.11 | 5.58 | 1.74 | 1.24 | 4.02 |
| 79 | M | 118 | 40 | 7.79 | 2.97 (2.81 - 3.17) | 1.91 | 2.23 | 1.61 | 3.17 | 3.02 |
| 72 | M | 173 | 49 | 4.34 | 1.71 (1.51 – 1.78) | 4.65 | 9.20 | 1.61 | 3.49 | 8.85 |
| 52 | M | 119 | 43 | 7.19 | 2.47 (2.03 - 4.50) | 1.06 | 4.57 | 1.37 | 1.74 | 4.46 |
| 80 | M | 176 | 39 | 5.36 | 1.61 (1.51 – 1.69) | 2.03 | 12.5 | 1.61 | 4.46 | 4.06 |
| 82 | F | 146 | 54 | 4.67 | 1.72 (1.47 - 1.83) | 6.47 | 4.60 | 1.94 | 6.73 | 2.54 |
| 74 | M | 121 | 44 | 6.91 | 1.77 (1.74 – 1.86) | 2.10 | 6.37 | 1.69 | 2.43 | 3.45 |
| 77 | F | 166 | 49 | 4.52 | 1.51 (1.45 - 1.56) | 3.65 | 14.4 | 1.61 | 3.99 | 7.88 |
| 79 | M | 158 | 39 | 5.97 | 1.81 (1.65 – 1.86) | 3.38 | 5.48 | 1.43 | 6.50 | 3.29 |
| 78 | M | 145 | 35 | 7.25 | 1.60 (1.48 - 1.77) | 3.40 | 4.72 | 1.60 | 2.53 | 4.81 |
| 73 | F | 156 | 41 | 5.75 | 1.90 (1.78 – 1.97) | 2.43 | 7.77 | 1.59 | 1.16 | 11.6 |
| 58 | F | 143 | 50 | 5.14 | 1.43 (1.40 - 1.51) | 6.46 | 6.92 | 1.57 | 3.60 | 6.83 |
| 73 | M | 163 | 36 | 6.27 | 2.76 (2.55 – 3.05) | 2.86 | 1.86 | 1.64 | 2.21 | 4.64 |
| 73 | M | 145 | 40 | 6.34 | 1.86 (1.72 - 1.93) | 2.53 | 5.04 | 1.53 | 2.47 | 4.94 |
| 75 | M | 108 | 38 | 8.96 | 1.82 (1.70 – 2.33) | 3.64 | 1.94 | 1.74 | 3.45 | 1.71 |
| 67 | F | 152 | 62 | 3.90 | 1.40 (1.32 - 1.61) | 9.22 | 8.86 | 1.55 | 9.09 | 5.62 |
| 54 | M | 147 | 38 | 6.58 | 1.74 (1.55 – 1.98) | 1.41 | 10.2 | 1.42 | 1.38 | 7.42 |
| 83 | M | 135 | 39 | 6.99 | 1.71 (1.66 - 1.91) | 2.93 | 5.47 | 2.00 | 2.89 | 2.75 |
| 79 | F | 197 | 50 | 3.73 | 1.77 (1.67 – 1.79) | 4.53 | 11.1 | 1.91 | 3.81 | 7.48 |
| 81 | M | 139 | 35 | 7.56 | 1.72 (1.72 - 2.37) | 5.08 | 2.35 | 1.64 | 4.28 | 2.09 |
| 49 | M | 115 | 42 | 7.61 | 1.69 (1.46 – 1.72) | 1.11 | 11.0 | 1.62 | 1.33 | 6.25 |
| 79 | M | 146 | 35 | 7.20 | 1.76 (1.69 - 1.87) | 4.48 | 2.42 | 1.49 | 3.58 | 2.94 |

E indicates elastic modulus, SE, strain energy.

1. Specimen thickness shows the median and interquartile range of ten measurements by a digital caliper.
2. Aortic wall thickness is the mean of five measurements in a cross-section in EGCT image.
