# Supplementary material for: In vivo aortic elasticity measurement using electrocardiogram-gated computed tomography: validation with ex vivo loading test
Source: Interdiscip Cardiovasc Thorac Surg. 2025 Aug 19;40(8):ivaf148. doi: 10.1093/icvts/ivaf148 (PMC12375406; doi:10.1093/icvts/ivaf148)
Supplement: ivaf148_Supplementary_Data [file ivaf148_supplementary_data.zip › Supplemental_figure/Supprementary table 2.docx]

Supplementary Table 2. Measurements by loading test and ECG-gated CT images

| Subgroup | Classification criteria | n | Loading test | | | | ECG-gated CT | | | |
| --- | --- | --- | --- | --- | --- | --- | --- | --- | --- | --- |
|  |  |  | Elastic modulus  [MPa] | p | Strain energy  [kJ/m^3^] | p | Elastic modulus  [MPa] | p | Strain energy  [kJ/m^3^] | p |
| Aortic diameter | ≥ 45 mm | 19 | 3.82 (3.13-6.46) | .0010 | 7.16 (5.66-11.1) | .0236 | 3.60 (2.65-5.06) | .0404 | 5.62 (3.17-7.88) | .1379 |
|  | < 45 mm | 30 | 2.69 (1.77-3.58) |  | 5.53 (3.87-7.05) |  | 2.66 (1.46-4.20) |  | 3.94 (3.05-5.58) |  |
| Aortic wall thickness | < 1.71 mm | 24 | 3.64 (2.94-4.63) | .0188 | 7.04 (5.69-12.1) | .0006 | 3.25 (2.01-4.38) | .3556 | 5.72 (4.12-7.75) | .0112 |
|  | ≥ 1.71 mm | 25 | 2.53 (1.87-3.63) |  | 5.04 (2.40-6.60) |  | 2.65 (1.58-4.23) |  | 3.65 (2.98-4.64) |  |
| Systolic blood pressure | < 144 mmHg | 24 | 2.87 (1.47-3.74) | .0482 | 5.52 (2.87-6.76) | .0276 | 2.72 (1.69-3.42) | .0396 | 4.15 (2.82-6.03) | .1319 |
|  | ≥ 144 mmHg | 25 | 3.56 (2.52-4.54) |  | 7.77 (5.26-10.6) |  | 3.81 (2.34-4.82) |  | 4.81 (3.18-7.68) |  |
| Age | < 70 | 17 | 2.07 (1.25-3.43) | .0216 | 6.92 (4.93-13.3) | .0568 | 1.73 (1.43-3.09) | .0032 | 5.62 (4.11-8.64) | .0101 |
|  | ≥ 70 | 32 | 3.59 (2.61-4.32) |  | 5.66 (4.04-7.62) |  | 3.68 (2.60-4.55) |  | 3.94 (2.80-5.59) |  |
| Sex | Male | 32 | 3.03 (1.86-3.74) | .0303 | 5.62 (4.30-7.08) | .0338 | 3.09 (1.74-4.25) | .5672 | 3.94 (3.03-4.84) | .0110 |
|  | Female | 17 | 3.65 (2.67-5.77) |  | 7.77 (4.29-13.6) |  | 3.17 (1.76-5.09) |  | 6.83 (3.90-9.41) |  |
| Anatomical characteristics  (aorta) | Aorta < 45 mm | 20 | 3.17 (2.09-3.75) | Ref | 5.17 (2.70-5.73) | Ref | 3.09 (2.28-4.25) | Ref | 3.47 (2.96-4.90) | Ref |
|  | AAE | 5 | 2.03 (1.26-3.40) | .4866 | 11.0 (6.26-15.0) | .1443 | 1.68 (1.36-5.94) | .9563 | 6.25 (2.81-10.6) | .4417 |
|  | ATAA | 19 | 3.82 (3.13-6.46) | .0721 | 7.16 (5.66-11.1) | .0107 | 3.60 (2.65-5.06) | .5680 | 5.62 (3.17-7.88) | .1392 |
|  | TBAD | 5 | 1.15 (1.09-2.27) | .0259 | 6.37 (5.08-7.08) | .3987 | 1.24 (1.09-2.08) | .0314 | 4.46 (3.73-8.24) | .3987 |
| Anatomical characteristics  (aortic valve) | Normal TAV | 27 | 2.86 (1.90-3.64) | Ref | 5.04 (3.14-6.37) | Ref | 3.45 (1.68-4.58) | Ref | 3.65 (2.94-4.63) | Ref |
|  | TAV post AVR | 14 | 3.74 (2.90-5.79) | .0729 | 7.47 (5.61-11.0) | .0134 | 3.11 (1.79-5.26) | .9791 | 6.70 (3.08-8.12) | .0216 |
|  | BAV | 8 | 3.30 (2.18-4.76) | .4724 | 11.6 (6.84-16.7) | .0006 | 3.01 (1.75-3.50) | .7341 | 7.72 (5.47-9.84) | .0003 |

Classification criteria for aortic wall thickness (1.71 mm) and blood pressure (144 mmHg) are their medians. AAE indicates annuloaortic ectasia; ATAA, ascending thoracic aortic aneurysm; AVR, aortic valve replacement; BAV, bicuspid aortic valve; ECG, electrocardiogram; TAV, tricuspid aortic valve; TBAD, type B aortic dissection.
