# Supplementary figures and images for: In vivo aortic elasticity measurement using electrocardiogram-gated computed tomography: validation with ex vivo loading test
Source: Interdiscip Cardiovasc Thorac Surg. 2025 Aug 19;40(8):ivaf148. doi: 10.1093/icvts/ivaf148 (PMC12375406; doi:10.1093/icvts/ivaf148)

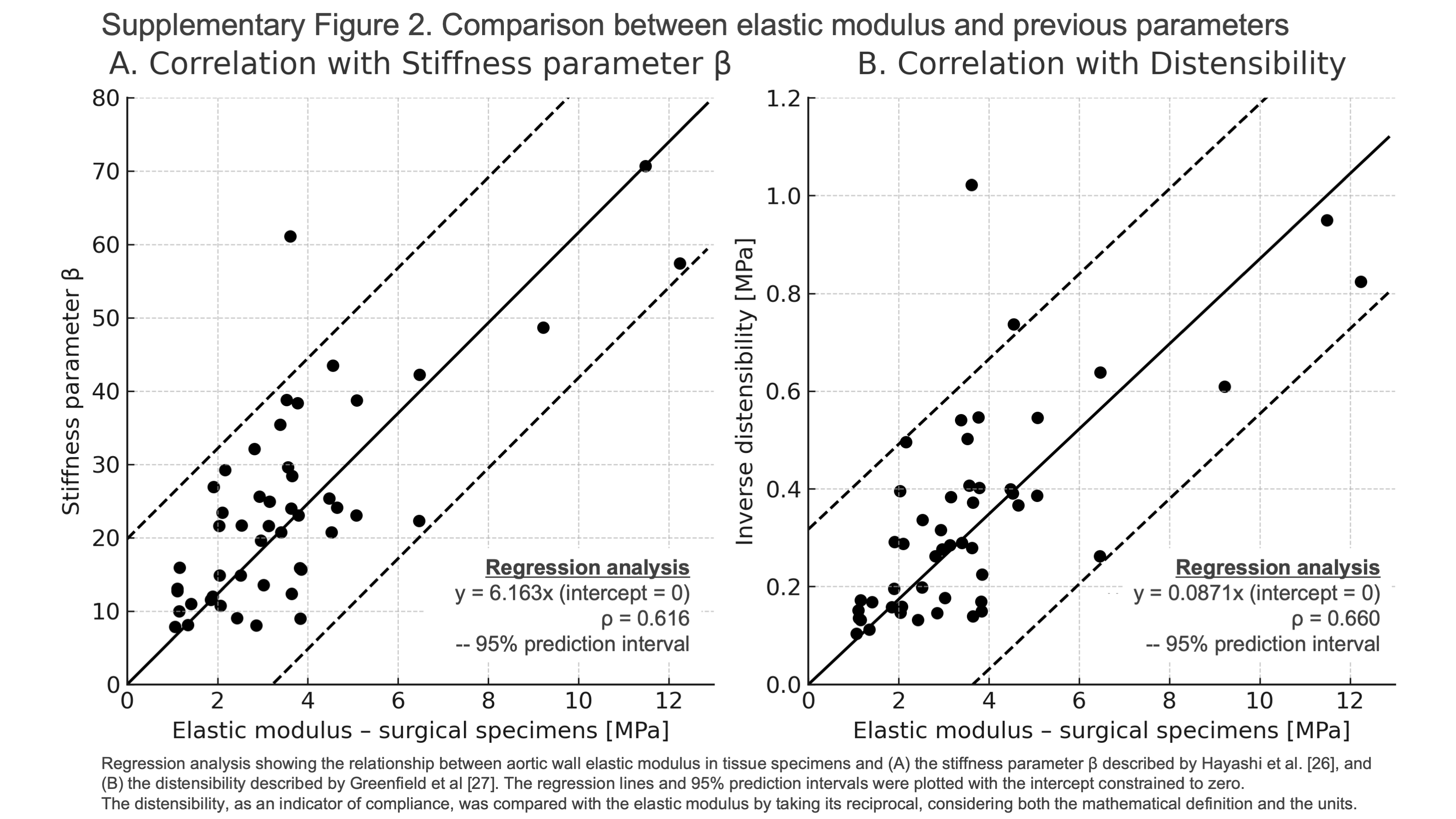

Supplement: ivaf148_Supplementary_Data [file ivaf148_supplementary_data.zip › Supplemental_figure/Supplementary figure 2legend.png]
